# Dietary modulation of the gut microbiota – a randomised controlled trial in obese postmenopausal women

**DOI:** 10.1017/S0007114515001786

**Published:** 2015-07-02

**Authors:** Lena K. Brahe, Emmanuelle Le Chatelier, Edi Prifti, Nicolas Pons, Sean Kennedy, Trine Blædel, Janet Håkansson, Trine Kastrup Dalsgaard, Torben Hansen, Oluf Pedersen, Arne Astrup, S. Dusko Ehrlich, Lesli H. Larsen

**Affiliations:** 1 Department of Nutrition, Exercise and Sports, Faculty of Science, University of Copenhagen, Rolighedsvej 26, 1958Frederiksberg C, Denmark; 2 INRA, Institut National de la Recherche Agronomique, US 1367 Metagenopolis, Jouy-en-Josas, France; 3 Arla Strategic Innovation Centre, Stockholm, Sweden; 4 Department of Food Science, Faculty of Science and Technology, Aarhus University, Aarhus, Denmark; 5 Novo Nordisk Foundation Centre for Basic Metabolic Research, University of Copenhagen, Copenhagen, Denmark

**Keywords:** Gut microbiota, Probiotics, Flaxseed mucilage, Obesity-related disease, Metagenomics

## Abstract

The gut microbiota has been implicated in obesity and its progression towards metabolic disease. Dietary interventions that target the gut microbiota have been suggested to improve metabolic health. The aim of the present study was to investigate the effect of interventions with *Lactobacillus paracasei* F19 or flaxseed mucilage on the gut microbiota and metabolic risk markers in obesity. A total of fifty-eight obese postmenopausal women were randomised to a single-blinded, parallel-group intervention of 6-week duration, with a daily intake of either *L. paracasei* F19 (9·4 × 10^10^ colony-forming units), flaxseed mucilage (10 g) or placebo. Quantitative metagenomic analysis of faecal DNA was performed to identify the changes in the gut microbiota. Diet-induced changes in metabolic markers were explored using adjusted linear regression models. The intake of flaxseed mucilage over 6 weeks led to a reduction in serum C-peptide and insulin release during an oral glucose tolerance test (*P*< 0·05) and improved insulin sensitivity measured by Matsuda index (*P*< 0·05). Comparison of gut microbiota composition at baseline and after 6 weeks of intervention with flaxseed mucilage showed alterations in abundance of thirty-three metagenomic species (*P*< 0·01), including decreased relative abundance of eight *Faecalibacterium* species. These changes in the microbiota could not explain the effect of flaxseed mucilage on insulin sensitivity. The intake of *L. paracasei* F19 did not modulate metabolic markers compared with placebo. In conclusion, flaxseed mucilage improves insulin sensitivity and alters the gut microbiota; however, the improvement in insulin sensitivity was not mediated by the observed changes in relative abundance of bacterial species.

More than half a billion people are obese worldwide, causing an estimated 2·8 million deaths each year due to metabolic comorbidities, such as CVD and type 2 diabetes (T2D)^(^ ^1^ ^)^. Metagenomic studies have suggested that obesity-related metabolic diseases are accompanied by alterations in gut bacteria gene composition and abundance^(^ ^2^ ^–^ ^7^ ^)^. Noticeably, microbial genes seem to be a stronger predictor of T2D than common anthropometric risk markers^(^ ^7^ ^)^ and variation in the human genome^(^ ^3^ ^)^, as shown in European and Chinese individuals, respectively, although the most discriminatory microbial genes differed between the Chinese^(^ ^3^ ^)^ and European individuals^(^ ^7^ ^)^.

Still, it is not clear whether shifts in the gut microbiota can cause metabolic diseases, or if they are just a consequence. However, causality is suggested by studies in germ-free mice^(^ ^8^ ^,^ ^9^ ^)^, and one study in human subjects^(^ ^10^ ^)^. These studies have shown that transplantation of gut microbiota can induce modifications in the microbiota of the receiving host, associated with either impaired or improved metabolic health, depending on the phenotype of the donor. Studies in human subjects that have measured diet-induced alterations in gut metagenomic and whole-body metabolic markers simultaneously have shown that modifications in the microbiota are accompanied by improvements in glucose homeostasis and lipid metabolism^(^ ^11^ ^,^ ^12^ ^)^. Together, these studies suggest that the gut microbiota constitute a promising target in the prevention of metabolic diseases.

Food components that directly target the gut microbiota include pre- and probiotics. Probiotics are living micro-organisms that, when ingested, provide health benefits, either directly through interactions with host cells or indirectly through effects on other bacterial species^(^ ^13^ ^)^. Common probiotics include *Lactobacillus* species^(^ ^14^ ^)^, and *Lactobacillus paracasei* has previously been associated with a healthy metabolic profile^(^ ^15^ ^,^ ^16^ ^)^. Prebiotics are dietary fibres that are selectively fermented by the gut bacteria and induce specific changes in the composition and/or activity of the gut microbiota that provide benefits to host health^(^ ^17^ ^)^. Flaxseed (*Linum usitatissimum* L.) contains approximately 30 % of dietary fibres, of which one-third are soluble viscous fibres (mucilage)^(^ ^18^ ^)^ that induce beneficial effects on glucose homeostasis and lipid metabolism in human subjects^(^ ^19^ ^–^ ^21^ ^)^. Flaxseed fibres have been shown to be highly fermentable in rats^(^ ^22^ ^)^; however, it is not known whether beneficial metabolic effects of flaxseed mucilage on human subjects can be explained by modulation of the gut microbiota.

The aim of the present study was to explore the effect of dietary interventions either with *L. paracasei* F19 or with flaxseed mucilage on gut microbiota and metabolic risk markers in obese postmenopausal women.

## Materials and methods

**Fig. 1:**
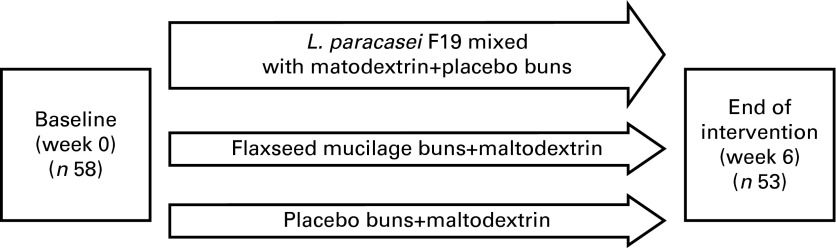
Illustration of the 6-week parallel-group intervention, with participants randomised to one of the three different diet groups.

A total of fifty-eight women were randomised to a parallel-group intervention of 6 weeks' duration with a daily intake of *L. paracasei* ssp. *paracasei* F19, flaxseed mucilage or placebo (Fig. 1). The participants were blinded to their allocation; *L. paracasei* F19 was mixed with maltodextrin and administered in sachets and the indistinguishable placebo product was administered in sachets with a corresponding dose of maltodextrin. Flaxseed mucilage was administered in breakfast buns and the corresponding placebo products were buns without flaxseed mucilage.

The primary outcomes were the effect on the gut microbiota composition and insulin sensitivity; the secondary outcomes were the effect on the inflammatory markers, blood lipids and fat mass distribution. Enrolment of participants was performed continuously by the same two researchers, and randomisation was carried out continuously using pre-prepared opaque sealed envelopes with an equal allocation ratio of 1:1:1. Sample size calculations were based on preliminary results from a subgroup of Danish participants in the Metagenomics of the Human Intestinal Tract (MetaHIT) study^(^ ^5^ ^)^. With a sample size of eighteen participants per group, a statistical power of 80 %, a two-sided significance level of 0·05 and with the assumption of equal variances, the study will identify the changes between independent groups in gut microbiota composition corresponding to 1 sd, e.g. a difference of 40 000 gene counts will be identified if the sd is 40 000 gene counts, and similarly, a 0·5 difference in insulin sensitivity (homeostatic model assessment of insulin resistance, HOMA-IR) will be identified for an sd of 0·5.

The participants attended a screening visit within the 2 weeks before randomisation and four visits after enrolment: visit 1 at baseline (day 0); visit 2 at day 28 of the intervention; visit 3 at the end of the intervention (day 42) and visit 4 4 weeks after the end of the intervention. After the completion of the study, the participants were offered a 10-week weight reduction programme. During the visits, blood was drawn, faecal samples were collected, body composition and blood pressure were measured, and information on dietary intake, physical activity level and adverse events were obtained. The present analyses included the 6-week intervention period that was the primary outcome of the study.

### Study population

Participants were recruited from the Copenhagen area through advertisements in newspapers and relevant web sites. The inclusion criteria were women aged 40–70 years, ≥ 1 year since last menstruation, BMI of 30–45 kg/m^2^, waist circumference >80 cm and leucocyte blood count >4·7 × 10^9^ cells/l. The exclusion criteria were gastrointestinal diseases, chronic diseases (such as type 1 diabetes or liver cirrhosis), medically treated T2D or dyslipidaemia, intake of antibiotics within the previous 3 months, intake of supplementary pro- or prebiotics or abnormal high quantities of fermented foods (>400 g/d) in the previous 6 weeks, or inability to comply with the research protocol. The present study took place at the Department of Nutrition, Exercise and Sports, Faculty of Sciences, University of Copenhagen, Denmark, from September 2011 to September 2012.

The present study was conducted according to the guidelines laid down in the Declaration of Helsinki, and all procedures involving human participants were approved by the Ethics Committee for the Capital Region of Denmark (journal H-3-2011-067). Written informed consent was obtained from all participants. The present study was registered at ClinicalTrial.gov (NCT01433120).

### Intervention

The probiotic product contained *L. paracasei* F19 (9·4 × 10^10^ colony-forming units/dose). The probiotic and the placebo (pure maltodextrin) sachets were indistinguishable. The products were stored at − 80°C until the weekly supply to the participants, after which they were stored in their personal freezer at − 18°C until consumption. Participants dissolved the entire content of the sachet in a glass of water and consumed it in the morning. Bacterial viability was confirmed at the end of the intervention by anaerobic culture tests of a sample of the products. Dilution of samples was spread on deMan, Rogosa and Sharpe (MRS) pH 5·4 agar. Plates were incubated anaerobically 72 h at 37°C. All characteristic colonies were checked by microscopy and counted (Arla Foods amba).

Flaxseed mucilage was extracted from whole flaxseed under heat treatment (Biogin Biochemicals Company Limited). Breakfast buns based on wheat with and without flaxseed mucilage were produced by the Department's experimental kitchen, and the daily amount of flaxseed mucilage administered to the active group was 10 g (5 g/bun). The products were stored at − 18°C at the Department until the weekly supply to the participants. The intervention and placebo buns were comparable in visual appearance, taste and macronutrient composition. They were consumed during the morning hours. The participants were otherwise instructed to maintain habitual dietary habits.

Participants were required to keep a diary of their intake of study products, and these were collected at the weekly visits at the Department. A compliance score was calculated as the percentage of the prescribed test products a participant reported to have consumed during the 6 weeks of intervention. The participants were interviewed about all types of potential adverse effects at each visit by the use of broad, open-ended questions; in addition, they were asked specifically to changes in stool characteristics (consistency and frequency).

### Body composition, dietary and activity records

Body composition was assessed by dual-energy X-ray absorptiometry (iDXA; Lunar Radiation Company), BMI (kg/m^2^), waist circumference and intra-abdominal adipose tissue (cm^2^: − 208·2+4·62 (sagittal diameter, cm)+0·75 (age, years)+1·73 (waist, cm)+(0·78 (trunk fat, %)))^(^ ^23^ ^)^. Registration of 3 d weighed dietary intake and physical activity level were performed within the week before each visit. A registered dietitian analysed all dietary records using the Danish dietary software program (Dankost Pro). Physical activity was registered using the Physical Activity Scale^(^ ^24^ ^)^ where the metabolic equivalent value is calculated based on the time spent on nine different intensity levels ranging from sleep to very strenuous activities during 24 h.

### Biochemical analyses

Fasting blood samples were obtained after an overnight fast followed by an oral glucose tolerance test (OGTT) where 75 g of glucose were dissolved in 300 ml of water and consumed within 5 min. During the OGTT, blood samples were taken with 30 min intervals for 3 h to ensure return to baseline values. Blood for glucose analysis was collected in iced sodium fluoride tubes. Blood samples for the analysis of leucocytes count, high-sensitivity C-reactive protein (hsCRP), lipopolysaccharide-binding protein (LBP), IL-6 and angiopoietin-like protein 4 (ANGPTL4) were drawn in iced EDTA tubes. Blood samples for all other analyses were collected in non-coated tubes. Blood samples were centrifuged for 10 min at 2500 ***g*** at 4°C and kept at − 80°C until analyses were performed. Insulin resistance was assessed by glucose, insulin and C-peptide at the fasting and stimulated state. Inflammatory state was assessed by leucocytes count, hsCRP, TNF-α, IL6 and LBP. Lipid metabolism was assessed by measurement of serum concentrations of total cholesterol, LDL-cholesterol, HDL-cholesterol and TAG, and plasma concentration of ANGPTL4. ABX Pentra 400 (Horiba ABX) was used to analyse glucose (intra- and inter-assay CV: 1·1 and 1·5 %), TAG (intra- and inter-assay CV: 3·8 and 3·0 %), total cholesterol (intra- and inter-assay CV: 1·0 and 1·7 %), HDL-cholesterol (intra- and inter-assay CV: 1·2 and 2·7 %), LDL-cholesterol (intra- and inter-assay CV: 1·3 and 3·3 %) and hsCRP (intra- and inter-assay CV: 3·6 and 6·5 %). Immulite 1000 (Siemens Medical Solutions Diagnostics) was used to analyse insulin (intra- and inter-assay CV: 4·2 and 7·3 %) and C-peptide (intra- and inter-assay CV: 6·9 and 5·2 %). Sysmex KX-21 hematology (Sysmex GmbH) was used to analyse the leucocytes count (intra- and inter-assay CV: 2·0 and 1·4 %). LBP (intra- and inter-assay CV: 6·1 and 9·8–17·8 %) was analysed with a human LBP Elisa kit (Abnova). TNF-α (intra- and inter-assay CV: 5·4 and 6·1 %), IL-6 (intra- and inter-assay CV: 7·4 and 8·5 %) and ANGPTL4 (intra-assay CV: 3·8 %) were analysed with ELISA (antibodies were purchased from R&D Systems). Insulin resistance was estimated by HOMA-IR^(^ ^25^ ^)^. Data from the OGTT were evaluated by AUC analysis and by the Matsuda index^(^ ^26^ ^)^.

### Stool samples and microbiota analyses

Participants collected samples of faeces in two 20 ml tubes (samples A and B) within 2 d before the visit. The samples were either stored immediately at − 80°C or briefly stored in personal − 18°C freezers before transport to the laboratory in cooled containers. Total faecal DNA from the fifty-three participants who completed the dietary intervention was extracted, sequenced and analysed by quantitative metagenomics at Metagenopolis (INRA). To obtain homogenous representation of all bacterial species, DNA extraction involved use of quenching solutions that protect DNA potentially prematurely released by lysis of fragile cells from degradation by DNases present in the stools and a bead-beating step that assures lysis of particularly robust cells^(^ ^27^ ^,^ ^28^ ^)^. A Barcoded Fragment Library was prepared for each sample and DNA sequencing data were generated using the SOLiD 5500xl sequencers (Life Technologies). An average of 65 (sd 48) million 50-base-long single reads was determined for each sample.

Primary data analyses, from reads to generation of a raw count matrix, were performed using METEOR Studio pipeline for quantitative metagenomic profiling developed at INRA MetaGenoPolis based on the iMOMi database. Reads generated from the SOLiD sequencer were trimmed to thirty-five bases and then mapped on the reference catalogue of 3·3 million genes^(^ ^29^ ^)^ using Bowtie software with a maximum of three mismatches and selection of the best hit. If multiple alignments were found, counts were divided equally between the aligned genes. Using both thirty-five bases reads and three maximum mismatches allows to take into account the strain variability and the non-redundant nature of the gene catalogue where redundant open reading frames were removed using a criterion of 95 % identity over 90 % of the shorter open reading frame length. Average of 38 (sd 14) million reads per sample was thus mapped and used to construct a raw gene count matrix.

Secondary analyses, from matrix normalisation to microbiota analyses, were performed using MetaOMineR package, an analytical suite (R language) developed at the INRA MetaGenoPolis^(^ ^4^ ^,^ ^5^ ^)^ that contains different algorithms and routines to normalise and analyse raw gene count matrixes to extract biological signals. To decrease technical bias due to different sequencing depth, 24 million reads were randomly selected for each sample using a draw without replacement. Abundance of each gene in a sample was normalised by dividing the number of reads that uniquely mapped to a gene by its nucleotide length. Then, normalised gene abundance was transformed in to frequencies by dividing with 24 million (see online Supplementary Fig. S1). The resulting set of gene frequencies, the microbial gene profile of an individual, was used for further analyses.

Microbial gene richness was measured by counting the number of genes that are present for a given sample using a downsized count matrix as performed in the original studies^(^ ^4^ ^,^ ^5^ ^)^. To be able to compare the present study and the former ones, we used the same method, estimating gene count richness using at 11 million unique reads matrix and a gene richness categorical variable computed by applying a threshold of 480 000 bacterial genes, to distinguish low from high gene count^(^ ^4^ ^,^ ^5^ ^)^. A prediction model for bacterial richness based on the six bacterial species was applied in a receiver operating characteristic analysis as described previously^(^ ^5^ ^)^, computing the sum of mean abundance of species with greater abundance in high gene count than in low gene count minus the sum of those with greater abundance in low gene count than in high gene count. Richness was estimated using either shared or unique matrix downsized matrices, and either gene count for richness or richness index such as exponential of Shannon richness index or inverse Simpson index.

Differentially abundant genes between both time points were selected for each diet using paired Wilcoxon test (*P*< 0·01), then clustered into metagenomic species (MGS), using the method based on binning co-abundant genes across all individuals samples reported previously^(^ ^5^ ^)^. To verify that the genes from a given cluster belonged to the same genome and to annotate the MGS taxonomically, we performed blastN and blastP analyses using a collection of 6006 genomes (the available reference genomes from the National Center for Biotechnology Information and the set of draft gastrointestinal genomes from the Data Analysis and Coordination Center of the HMP and MetaHIT (3 August 2012 version)). MGS were assigned to a given genome when more than 80 % of the genes matched the same genome using blastN, at a threshold of 95 % identity over 90 % of gene length. The remaining MGS were annotated using blastP analysis and assigned to a given taxonomical level from genus to super kingdom level if more than 80 % of their genes had the same level of assignment. Data were indexed in a relational database permitting downstream access to genes, gene function, phylogeny and covariation analysis.

### Quantification of SCFA using the ethyl chloroformate-NEFA method

For the quantification of SCFA, approximately 10 g of stool were mixed with an equal amount of deionised H_2_O (18·2 MΩ) filtered water (0·22 mm). Sample preparation, analyses by the ethyl chloroformate NEFA method and GC, were performed by applying methods described in detail by Amer *et al.* ^(^ ^30^ ^)^. Quantification of ethyl chloroformate-NEFA esters was obtained using external calibration curves for each NEFA. The two isotopic standards butyric internal standard (D7, 98 %) and Cambridge Isotope Laboratories, Inc. were used as internal standard for butyric acid.

### Statistical analyses

Effect of dietary interventions on biochemical and anthropometric parameters was analysed for completers by multiple linear regression models adjusted for baseline values, with stepwise backward elimination of the following covariates: age; body fat percentage (baseline); energy intake in the week preceding the last visit; changes in weight and physical activity level during the intervention. Log-transformation was applied to non-normally distributed variables. Comparisons of two means were performed using *t* tests. Regression coefficients, CI and *P*-values are reported from the adjusted regression models, while non-adjusted mean values for changes in biochemical parameters during the intervention are reported in Table 2. Correlation analyses between bacterial gene count and biochemical measures were analysed by Spearman's rank correlation coefficient. At an explorative level, the potential effect of diet-induced compositional changes in the gut microbiota on metabolic markers was examined by separate analyses where MGS that changed during the intervention were included as explanatory variables in the multiple regression models. Statistical significance was considered at a threshold value of 0·05. Analyses were performed using MetaOMinerR package (developed at Metagenopolis, INRA) and JMP version 9.0.2 (SAS Institute, Inc.).

## Results

**Fig. 2:**
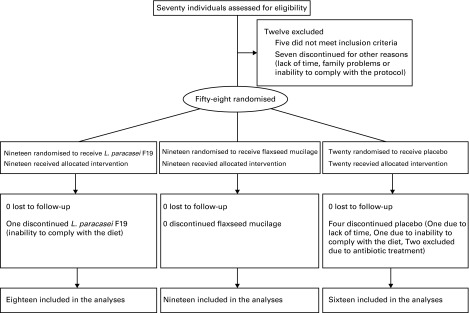
Flow chart of the study.

A total of fifty-three participants completed the study (91 %). Dropout rates were not significantly different between groups, and no significant differences were found between dropouts and completers in anthropometric and biochemical characteristics at baseline. Details on recruitment, randomisation and study flow are shown in Fig. 2. Baseline characteristics for all the participants by intervention group are presented in Table 1. There were no differences in the anthropometric measures between groups; however, the participants in the *L. paracasei* F19 group had significantly impaired insulin sensitivity compared with the flaxseed mucilage group, and a significantly lower bacterial gene count compared with the placebo group. All the participants reported to have consumed at least 75 % of the study products during the 6 weeks of intervention, and the mean compliance score was above 90 % in all the groups.

**Table 1:** Baseline characteristics presented by diet group (Mean values and standard deviations)

	*Lactobacillus paracasei* F19 (*n* 19)	Flaxseed mucilage (*n* 19)	Placebo (*n* 20)
	Mean	sd	Mean	sd	Mean	sd
Age (years)	61·4	6·5	60·6	6·4	58·5	5·3
Height (cm)	163	5·8	164	7·0	165	5·7
Weight (kg)	91·1	9·4	93·8	7·8	93·8	11·5
BMI (kg/m^2^)	34·2	3·1	35·2	4·5	34·3	3·8
Waist circumference (cm)	105	6·8	104	9·2	104	11·0
IAAT (cm^2^)	172	21·2	171	28·6	169	33·4
Body fat (%)	46·7	4·1	48·0	3·3	46·8	3·6
Energy intake (kJ/d)	7824	1962	6859	1922	8153	1121
Protein (E%)	18·4	3·0	19·4	4·9	19·2	2·6
Carbohydrates (E%)	41·5	5·6	39·7	8·6	41·6	6·0
Fat (E%)	34·7	6·5	35·2	6·9	36·4	5·9
24-h-MET-time	41·6	9·3	38·4	8·8	41·7	8·1
Fasting plasma glucose (mmol/l)	5·89	0·76	5·75	0·90	5·81	0·77
Fasting serum insulin (pmol/l)	113	55	71	32	89	56
Fasting serum C-peptide (pmol/l)	958	344	713	185	816	385
AUC glucose (mmol/l per min)	7·6	1·7	7·0	1·7	6·8	1·0
AUC insulin (pmol/l per min)	476	212	331	154	420	303
AUC C-peptide (pmol/l per min)	3056	966	2466	754	2714	1204
HOMA-IR	5·11	2·91	3·16	1·76	3·94	2·59
Matsuda index	1·79	1·02	2·82	1·65	2·65	1·89
Serum total cholesterol (mmol/l)	6·13	1·06	6·36	0·89	5·76	0·69
Serum HDL-cholesterol (mmol/l)	1·46	0·26	1·40	0·22	1·56	0·42
Serum LDL-cholesterol (mmol/l)	3·73	0·84	4·11	0·84	3·44	0·74
Serum TAG (mmol/l)	1·69	0·83	1·51	0·77	1·07	0·32
Plasma ANGPTL4 (ng/ml)	8·16	3·08	7·77	3·06	7·59	1·36
Leucocyte count (10^9^/l)	5·55	1·05	5·46	0·92	5·60	1·64
Plasma hsCRP (mg/l)	2·90	3·78	3·73	3·69	4·14	2·90
Serum TNF-α (pg/ml)	2·66	3·53	2·64	1·60	1·81	0·70
Plasma IL-6 (pg/ml)	1·40	0·43	2·69	3·54	1·82	0·92
Plasma LBP (μg/l)	18·7	5·9	19·6	7·2	18·5	7·6
Total faecal SCFA (μg/kg)*	1901	1067	1556	1006	1692	869
Faecal butyric acid (μg/kg)*	917	632	892	508	907	443
Bacterial gene count*	556 338	144 657	618 100	114 608	646 688	77 872
Exponential Shannon index*	139 343	59 749	176 741	59 046	179 518	53 269
Inverse Simpson index*	44 335	25 089	55 039	24 854	55 654	23 140

IAAT, intra-abdominal adipose tissue; E%, energy percentage; 24-h-MET-time, 24-h metabolic equivalent value; HOMA-IR, homeostatic model assessment of insulin resistance; ANGPTL4, angiopoietin-like protein 4; hsCRP, high-sensitivity C-reactive protein; LBP, lipopolysaccharide-binding protein.

* The analyses are only performed in fifty-three completers.

### Diet-induced changes in biochemical markers

Multiple linear regression analyses of the effect of dietary intervention on biochemical markers showed that the intake of flaxseed mucilage over 6 weeks improved insulin sensitivity as shown by a decrease in the serum C-peptide and insulin response following the OGTT. The AUC values for serum C-peptide and insulin were 337 pmol/l per min (95 % CI 168·7, 505·1) and 65·9 pmol/l per min (95 % CI 26·5, 105·2) lower compared with placebo (adjusted values), corresponding to 12 and 13 % improvement within the flaxseed mucilage group (Table 2). Matsuda's index was increased by 0·4 (95 % CI 0·1, 0·7), compared with placebo (adjusted values); corresponding to 11 % increase within the flaxseed mucilage group. Markers for lipid metabolism and inflammation were reduced after 6 weeks in the flaxseed group (Table 2); however, there were no significant differences compared with placebo. There was no effect of *L. paracasei* F19 on insulin sensitivity, lipid metabolism, inflammatory markers or anthropometric measures compared with placebo (Table 2).

**Table 2:** Biochemical characteristics after 6 weeks of dietary intervention and changes from baseline (Mean values with their standard errors)

	*Lactobacillus paracasei* F19 (*n* 18)	Flaxseed mucilage (*n* 19)	Placebo (*n* 16)	
	Mean	se	Mean	se	Mean	se	*P* *
Fasting plasma glucose (mmol/l)	5·75	0·17	5·67	0·16	5·58	0·14	
Δ Glucose	− 0·16	0·083	− 0·083	0·082	− 0·30	0·14	0·524
Fasting serum insulin (pmol/l)	114	13	72	8·0	104	12	
Δ Insulin	− 0·65	5·75	0·52	4·69	9·05	6·76	0·166
Fasting serum C-peptide (pmol/l)	970	91	699	51	917	83	
Δ C-peptide	4·9	37·0	− 13·7	33·3	57·7	53·3	0·258
AUC glucose (mmol/l per min)	7·49	0·43	6·91	0·38	7·00	0·29	
Δ AUC glucose	− 0·035	0·17	− 0·20	0·15	0·091	0·16	0·523
AUC insulin (pmol/l per min)	504	52	297	32	481	71	
Δ AUC insulin	27	29	− 44	19	39	27	0·001
AUC C-peptide (pmol/l per min)	3175	252	2204	132	2866	283	
Δ AUC C-peptide	110	127	− 297	93	101	116	0·001
HOMA-IR	5·07	0·70	3·06	0·36	4·00	0·65	
Δ HOMA-IR	− 0·12	0·07	− 0·10	0·051	− 0·25	0·11	0·296
Matsuda index	1·81	0·24	3·05	0·40	2·17	0·36	
Δ Matsuda index	0·022	0·12	0·25	0·21	− 0·35	0·31	0·048
Serum total cholesterol (mmol/l)	6·24	0·28	5·89	0·21	5·72	0·17	
Δ Total cholesterol	0·12	0·18	− 0·47	0·13	− 0·11	0·13	0·154
Serum HDL-cholesterol (mmol/l)	1·45	0·056	1·34	0·047	1·54	0·089	
Δ HDL-cholesterol	− 0·0067	0·036	− 0·058	0·040	0·013	0·041	0·150
Serum LDL-cholesterol (mmol/l)	3·81	0·23	3·72	0·20	3·46	0·17	
Δ LDL-cholesterol	0·085	0·15	− 0·39	0·10	− 0·089	0·11	0·068†
Serum TAG (mmol/l)	1·96	0·22	1·48	0·12	1·16	0·079	
Δ TAG	0·24	0·14	− 0·028	0·14	0·13	0·057	0·205
Plasma ANGPTL4 (ng/ml)	7·80	0·67	6·92	0·60	7·19	0·47	
Δ ANGPTL4	− 0·25	0·33	− 0·85	0·46	− 0·69	0·52	0·142
Leucocyte count (10^9^/l).	5·41	0·25	5·14	0·26	5·50	0·34	
Δ Leucocyte count	− 0·18	0·17	− 0·33	0·22	0·07	0·15	0·290
Plasma hsCRP (mg/l)	3·43	0·95	2·68	0·80	2·89	0·66	
Δ hsCRP	0·43	0·26	− 0·91	0·20	− 1·25	0·45	0·036†
Serum TNF-α	2·79	0·78	2·60	0·43	1·63	0·14	
Δ TNF-α	0·056	0·13	− 0·043	0·15	− 0·011	0·16	0·419
Plasma IL-6	1·54	0·12	2·10	0·36	1·73	0·25	
Δ IL-6	0·10	0·091	− 0·59	0·49	− 0·16	0·16	0·644
Plasma LBP (μg/l)	19·7	1·5	18·1	1·5	16·3	1·5	
Δ LBP	0·56	0·82	− 1·51	0·84	− 1·88	0·93	0·221
Total faecal SCFA (μg/kg)	1730	224	1477	190	1685	231	
Δ SCFA	− 171	242	− 79	239	− 6·9	145	0·890
Faecal butyric acid (μg/kg)	789	128	806	93	837	89	
Δ Butyric acid	− 128	148	− 86	157	− 70	94	0·811

Δ, change during the 6 weeks of dietary intervention (unadjusted values); HOMA-IR, homeostatic model assessment of insulin resistance; ANGPTL4, angiopoietin-like protein 4; hsCRP, high-sensitivity C-reactive protein; LBP, lipopolysaccharide-binding protein.

* *P*-values are reported from linear regression models of the effect of dietary group on the specified parameters, when adjusted for relevant covariates including baseline values.

† The difference is to be found between the F19 and flaxseed mucilage group, but not when these are compared with placebo by Student's *t* test.

### Diet-induced changes in gut microbiota composition

Sequenced reads mapped against the MetaHIT gene catalogue of 3·3 million genes at a normal range of 61 (se 6) %. Of the fifty-three participants, forty-nine (92·5 %) had high gene count and four (7·5 %) had low gene count; two in the group allocated to *L. paracasei* F19 and two in the group allocated to flaxseed mucilage. A prediction model for bacterial richness based on six bacterial species, introduced by Cotillard *et al.* ^(^ ^4^ ^)^, was applied to the current data set and an AUC value of 0·94 was obtained.

At baseline, total bacterial gene count, which is a measure of gut bacterial richness, correlated negatively with total cholesterol (*r* − 0·28, *P*= 0·043), LDL-cholesterol (*r* − 0·30, *P*= 0·029), leucocytes count (*r* − 0·34, *P*= 0·015) and aspartate aminotransferase (*r* − 0·33, *P*= 0·018), and tended to correlate negatively with AUC glucose (*r* − 0·25, *P*= 0·069) and alanine aminotransferase (*r* − 0·24, *P*= 0·091).

Within the flaxseed mucilage group, bacterial gene count decreased with 47 271 genes over 6 weeks (95 % CI − 82 176, − 12 366, *P*= 0·011); however, this change was not significantly compared with placebo (*P*= 0·144). Bacterial gene count did not change within the *L. paracasei* F19 group (*P*= 0·473). The gene loss following intake of flaxseed mucilage for 6 weeks was confirmed by measures of α diversity; when evaluated by the exponential of Shannon diversity index and Simpson's inverse index, the decrease in diversity was 38 010 (95 % CI − 64 473, − 11 546, *P*= 0·007) and 17 515 (95 % CI − 30 992, − 4038, *P*= 0·014), respectively.

Comparison of microbiota composition at baseline and after 6 weeks of intervention with *L. paracasei* F19 showed alterations in faecal abundance of 2493 bacterial genes assigned to two MGS that increased during the intervention relative to baseline. These were identified as the species *Eubacterium rectale* and *Ruminococcus torques*. The proportion of total distributions of *E. rectale* recovered after analysis increased 3·3 times (*P*= 0·003) and *R. torques* increased 4·5 times (*P*< 0·001).

The placebo intervention led to altered faecal abundance of 7436 genes assigned to six MGS (*P*< 0·01), where the relative abundance of four MGS (*Roseburia hominis*, two Clostridiales and one unknown) decreased and the relative abundance of two MGS (*Eubacterium ventriosum* and one unknown) increased.

**Fig. 3:**
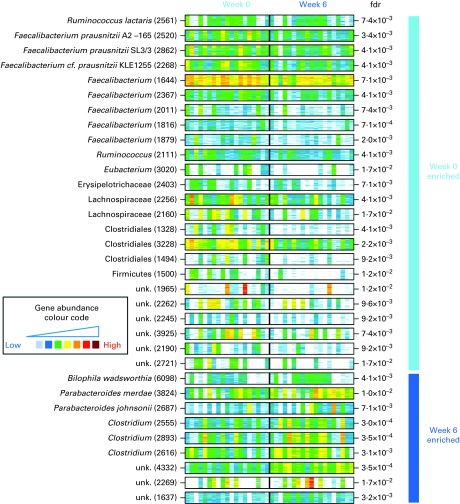
The presence and abundance of the thirty-three species that differed significantly before and after 6 weeks of intervention with flaxseed mucilage. Each barcode illustrates the abundance of a species, either enriched at baseline (top) or week 6 (bottom): samples are in columns (same order for both time points) and the fifty ‘tracer’ genes are in rows. Gene abundance is indicated by colour gradient from white (not detected) over blue, green and yellow to red (most abundant). Taxonomical information is given for each species; the number of genes within each metagenomic species is given in parenthesis. Fdr, Benjamini Hochberg multiple testing correction of paired Wilcoxon tests; unk., unknown taxonomy.

Comparison of microbiota composition at baseline and after 6 weeks of intervention with flaxseed mucilage showed alterations in faecal abundance of 41 090 bacterial genes assigned to thirty-three MGS, the relative abundance of twenty-four MGS decreased and nine MGS increased (Table 3 and Fig. 3). Of the thirty-three MGS that changed, twenty-four were identified at phylum level or below. Eight of the MGS identified as decreasing during the intervention were assigned to the *Faecalibacterium* genus, among which three *F. prausnitzii* species with sequence homology to the strains *F. prausnitzii* A2-165, *F. prausnitzii* SL3/3 and *F. cf. prausnitzii* KLE1255. The proportion of total distributions of these three species decreased 0·6 times (*P*< 0·01). Moreover, *Ruminococcus lactaris* decreased 0·9 times during the flaxseed mucilage intervention (*P*= 0·005).

**Table 3:** Gut bacteria changes in relative abundance after 6 weeks of intervention with 10 g flaxseed mucilage

Taxonomic level	*n*	Annotation
Decreased relative abundance		
Species	1	*Ruminococcus lactaris*
Species	3	*Faecalibacterium prausnitzii*
Genus	5	*Faecalibacterium*
Genus	1	*Ruminococcus*
Genus	1	*Eubacterium*
Family	1	Erysipelotrichaceae
Family	2	Lachnospiraceae
Order	3	Clostridiales
Phylum	1	Firmicutes
	6	Unknown
Increased relative abundance		
Species	1	*Bilophila wadsworthia*
Species	1	*Parabacteroides merdae*
Species	1	*Parabacteroides johnsonii*
Genus	3	*Clostridium*
	3	Unknown

Three of the nine MGS that increased in relative abundance after the flaxseed mucilage intervention were assigned to the *Clostridium* genus (Table 3 and Fig. 3). Furthermore, three MGS were identified to species level as *Bilophila wadsworthia*, *Parabacteroides merdae* and *Parabacteroides johnsonii*. The proportion of total distributions of these three species increased 2·6 times (*P*< 0·001), 3·6 times (*P*= 0·004) and 4·7 times (*P*< 0·001), respectively.

### Effect of gut microbiota on the improvement in insulin sensitivity

When exploring the role of relative changes in the gut microbiota on improvement in insulin sensitivity in the flaxseed mucilage group, the MGS that changed during the intervention were included in the multiple regression models in separate analyses. Relative changes in the MGS did not contribute to the effect of the flaxseed mucilage intervention on Matsuda index or AUC values for insulin and C-peptide, when separate analyses were performed where the delta values for the individual MGS were included in the model as explanatory variables.

### Adverse events

During the initial 4 weeks of the intervention, more adverse events were reported in the flaxseed group (*n* 11), compared with the placebo group (*n* 4; *P*= 0·023), while there was no difference in the occurrence of adverse events between the placebo and the *L. paracasei* F19 group (*n* 8; *P*= 0·297). The adverse events reported following the flaxseed intervention were increased flatulence and changed bowel habits with more frequent defecation and looser stool consistency. After 6 weeks, these symptoms were diminishing and only reported in seven participants, which was not different from the placebo group. There was no difference between the adverse events reported following the probiotic and placebo intervention that included both more frequent and less-frequent defecation. No serious adverse events were registered during the study.

## Discussion

The present study shows that daily intake of flaxseed mucilage over 6 weeks can improve insulin sensitivity and modify the gut microbiota in individuals with obesity. The relative decrease in the abundance of *F. prausnitzii* species following intake of flaxseed mucilage is surprising given the improvement in insulin sensitivity. Previous metagenomic studies have suggested that *F. prausnitzii* is more abundant in healthy individuals than in individuals with T2D^(^ ^3^ ^,^ ^7^ ^)^. In addition, prebiotics have been shown to induce a parallel increase in the faecal abundance of *F. prausnitzii* and improvement in insulin sensitivity in individuals with obesity^(^ ^11^ ^)^. A beneficial effect of *F. prausnitzii* on insulin sensitivity has been hypothesised to be due to its role as a major producer of the SCFA butyrate^(^ ^31^ ^)^, as butyrate seems to have an anti-inflammatory potential that might improve obesity-related metabolic complications such as insulin resistance^(^ ^32^ ^)^. The relative lower abundance of *F. prausnitzii* following intake of flaxseed mucilage is supported by the reduction observed in faecal butyrate content and by results from a previous study performed in rats, where fermentation of flaxseed fibres had been shown to yield a remarkably low proportion of butyrate, when compared with other dietary fibres^(^ ^22^ ^)^.

The improvement in insulin sensitivity following intake of flaxseed mucilage was accompanied by reductions in markers for inflammation and dyslipidaemia, although these changes were non-significant, compared with the placebo group. The gut microbiota has been implicated in the low-grade inflammation that characterises the progression from obesity to metabolic disease, as translocation of bacterial toxins, such as lipopolysaccharides, into the circulation can activate inflammatory pathways^(^ ^33^ ^,^ ^34^ ^)^. Impaired gut barrier function can be caused by adipocyte-derived inflammatory cytokines^(^ ^35^ ^)^, and increased translocation of lipopolysaccharides can be induced by a high-fat diet^(^ ^36^ ^,^ ^37^ ^)^. Thus, it is likely that the gut is an important mediator in obesity-related systemic inflammation. Prebiotic-induced modulation of the gut microbiota has been proposed to inhibit translocation of lipopolysaccharides based on observations in mice^(^ ^38^ ^)^; however, it is still unclear to what degree such mechanisms apply to human subjects. Since the decrease in LBP, hsCRP, TNF-α and IL-6 in the present study did not differ between the flaxseed mucilage and the placebo group, the improvement in insulin sensitivity did not appear to be mediated through inhibition of lipopolysaccharide translocation.

The design of the present study allowed us to explore whether the improvement in insulin sensitivity following intake of flaxseed mucilage could be due to a gut microbiota modulating effect. However, analyses showed that the changes in relative abundance of bacterial species induced by flaxseed mucilage did not explain any of the changes in the biochemical markers. When this is considered in relation to the flaxseed mucilage-induced changes in the microbiota that, based on the existing literature, are not in line with an improved host metabolic phenotype, we conclude that changes in the gut microbiota following intake of flaxseed mucilage do not appear to be a contributing factor to the beneficial effect of flaxseed mucilage on insulin sensitivity. Previously, it has been shown that daily intake of 5 g flaxseed mucilage reduced fasting blood glucose, total cholesterol and LDL-cholesterol in patients with T2D after 12 weeks^(^ ^20^ ^)^, and reduced total- and LDL-cholesterol in healthy individuals after 1 week^(^ ^21^ ^)^. In addition, acute meal tests with flaxseed mucilage have been shown to suppress postprandial blood glucose, insulin and lipid responses^(^ ^19^ ^,^ ^39^ ^)^. The beneficial metabolic effects of flaxseed mucilage observed in these previous studies and in the present study would then appear to be due to the ability of the soluble viscous fibres to delay gastric emptying and inhibit nutrient absorption rather than their ability to induce specific changes in the gut microbiota. Although, it cannot be excluded that the effect on insulin sensitivity was mediated through undetected bacterial species or changes in proteins and metabolites present in the gut that were not measured in the present study. The dominating molecular pathways in the bacterial species that increased during the flaxseed mucilage intervention are involved in pro-inflammatory signalling and managing of oxidative stress, pathways that have previously been suggested to be enriched in individuals with obesity-related metabolic diseases^(^ ^5^ ^)^. This shift towards a gut microbiome with a more pro-inflammatory potential might be explained by the fact that the flaxseed mucilage intervention provided more Cd in the diet. Despite that the Cd content in the flaxseed mucilage was at a concentration considered harmless to human health (0·113 mg/kg)^(^ ^40^ ^)^, it still might have induced modifications in the gut microbiome. This has been observed in previous studies in mice, where alterations were induced in the gut microbiome following administration of environmental relevant low doses of Cd in drinking-water over 3–8 weeks^(^ ^41^ ^–^ ^43^ ^)^.

This study showed a very limited effect of *L. paracasei* F19 (9·4 × 10^10^ colony-forming units/dose) on the gut microbiota and metabolic markers, which were non-significant compared with the placebo group. The storage of *L. paracasei* F19 was optimised to ensure that the probiotic strain was alive at ingestion. The diary kept by the participants indicated that they were highly compliant to the intervention.

A combined effect of this probiotic strain on both metagenomic and metabolic markers in human subjects has not been explored previously. In mice, *L. paracasei* F19 has been shown to affect the immune system and expression of genes involved in energy homeostasis and insulin sensitivity^(^ ^44^ ^)^, and to regulate body fat storage and lipoprotein metabolism^(^ ^45^ ^)^. Observational studies in human subjects have suggested that *L. paracasei* is associated with normal weight^(^ ^15^ ^)^ and negatively correlated with fasting blood glucose^(^ ^16^ ^)^ despite that several other *Lactobacillus* species have been suggested to be associated with obesity^(^ ^16^ ^,^ ^46^ ^,^ ^47^ ^)^ and T2D^(^ ^7^ ^)^. It is possible that we would have observed an effect of the probiotic intervention if it had been administered as part of a fermentable milk product, as a fermentable food matrix could potentially enhance probiotic efficacy^(^ ^48^ ^)^. A study among elderly has shown that daily intake of a fermented milk product with *L. paracasei* F19 (5·25 × 10^8^ colony-forming units) over 4 weeks stimulates the growth of other *Lactobacillus* species in the gut^(^ ^49^ ^)^. However, no effect on other *Lactobacillus* species was observed following 6 weeks of intake of *L. paracasei* F19 in the present study, although it cannot be excluded that the abundance of *Lactobacillus* species not included in the gene catalogue did change^(^ ^29^ ^)^.

The inclusion criteria in the present study were designed in order to recruit individuals at increased risk of metabolic diseases. Previous metagenomic studies have suggested that individuals with a metabolic risk profile are characterised by low gut microbiome richness^(^ ^4^ ^,^ ^5^ ^)^. The obese participants in these previous studies were comparable to the participants in the present study from a phenotype perspective; thus, it is surprising that only 8 % of the individuals in the present study had low gut microbiome richness as opposed to 23–40 % in the other cohorts^(^ ^4^ ^,^ ^5^ ^)^. The protocol for handling and analyses of microbiota samples were similar to the samples handling in the MetaHIT study^(^ ^5^ ^)^; however, it is possible that deviations in the prevalence of low microbiome richness between the cohorts can be explained by differences in habitual dietary habits. Yet, the negative correlations identified between bacterial gene count and metabolic risk markers support an association between gut microbiome richness and metabolic health. As does the lower microbiome richness detected at baseline in the participants allocated to *L. paracasei* F19, who were characterised by impaired insulin sensitivity, compared with the two other groups. This is interesting as the prediction model based on six bacterial species that has previously been demonstrated as a tool to identify individuals with low microbiome richness in two other obese cohorts^(^ ^4^ ^,^ ^5^ ^)^ also showed high specificity and sensitivity in this cohort.

In conclusion, the present study shows that intake of flaxseed mucilage improves insulin sensitivity and changes the gut microbiota in obese postmenopausal women, but suggests that the effect on insulin sensitivity is independent of the flaxseed mucilage-induced changes in abundance of bacterial species.

## Supplementary material

For supplementary material accompanying this paper visit http://dx.doi.org/10.1017/S0007114515001786. click here to view supplementary material
