# Supplementary figures and images for: Dietary modulation of the gut microbiota – a randomised controlled trial in obese postmenopausal women
Source: Br J Nutr. 2015 Jul 2;114(3):406–17. doi: 10.1017/S0007114515001786 (PMC4531470; doi:10.1017/S0007114515001786)

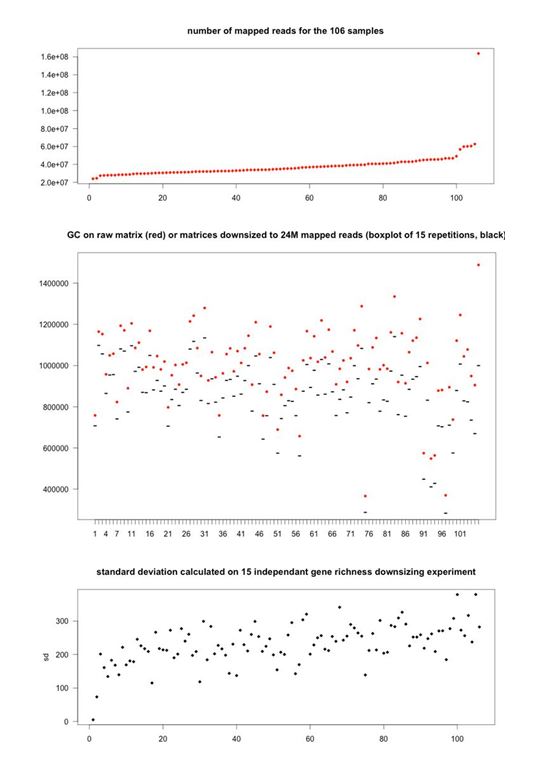

Supplement: Supplementary file 1 [file S0007114515001786sup001.jpg]
